# Supplementary figures and images for: Melatonin Action in Type 2 Diabetic Parotid Gland and Dental Pulp: In Vitro and Bioinformatic Findings
Source: Int J Environ Res Public Health. 2023 Sep 7;20(18):6727. doi: 10.3390/ijerph20186727 (PMC10530673; doi:10.3390/ijerph20186727)

Figure S1 HUMAN PAROTID GLAND CELLS

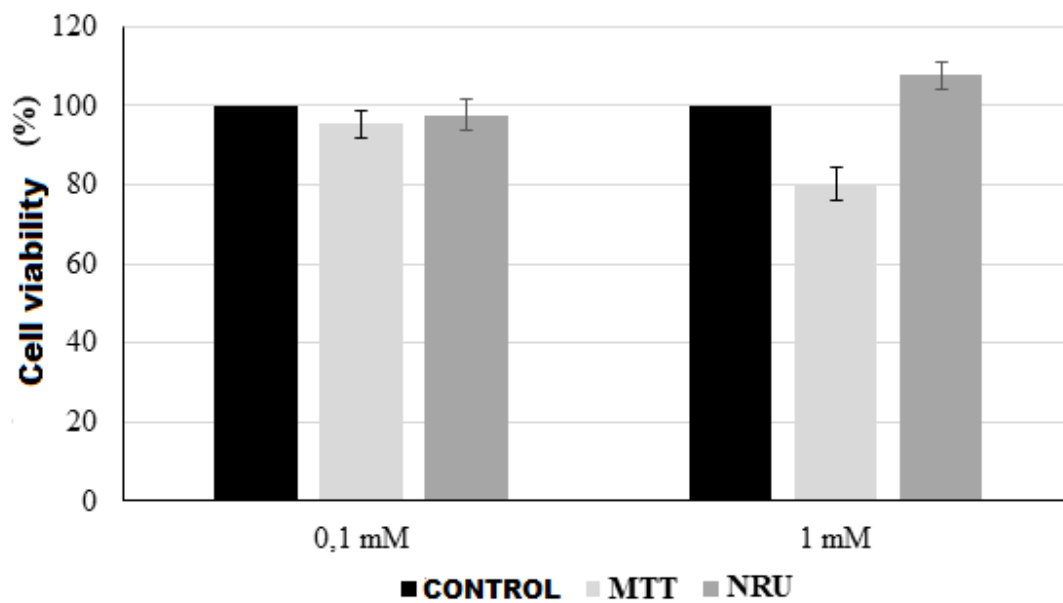

Figure S2 HUMAN DENTAL PULP CELLS

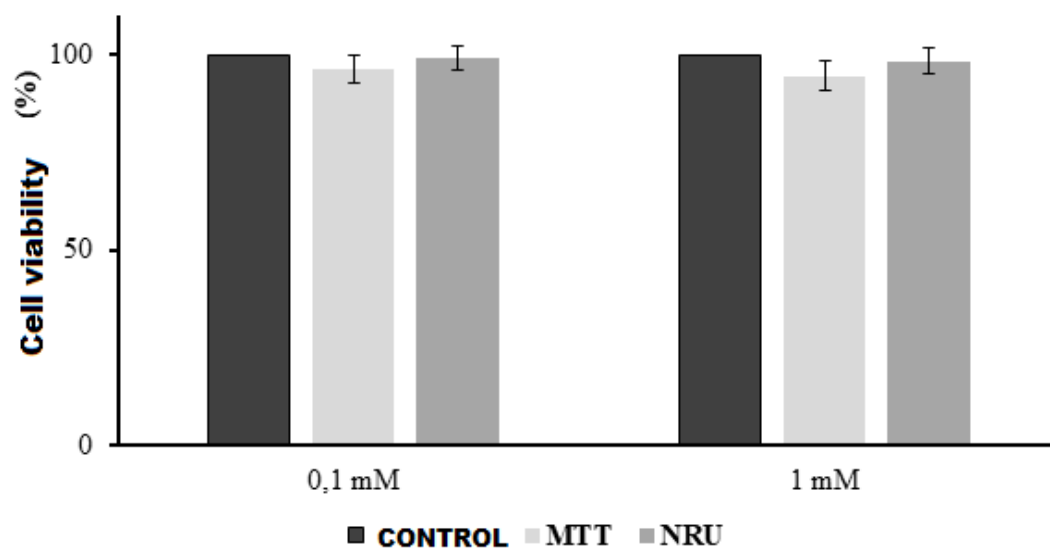

Supplement: Supplementary file 1 [file ijerph-20-06727-s001.zip › ijerph-2406042-supplementary.pdf]
